# Hypocellular acute myeloid leukemia with bone marrow necrosis in young patients: two case reports

**DOI:** 10.1186/1752-1947-3-27

**Published:** 2009-01-26

**Authors:** Deepali Jain, Tejinder Singh, Naresh Kumar

**Affiliations:** 1 Department of Pathology, Maulana Azad Medical College, New Delhi, 110002, India; 2 Department of Medicine, Maulana Azad Medical College, New Delhi, 110002, India

## Abstract

**Introduction:**

Hypocellular variants of acute myeloid leukemia are very rare and almost always occur in old aged patients. In contrast, hypocellular acute lymphoblastic leukemia usually occurs in children.

**Case presentation:**

We report two Indian patients with hypocellular acute myeloid leukemia, a 32-year-old woman and a 13-year-old boy. Interestingly, one of the patients also showed bone marrow necrosis.

**Conclusion:**

Hypocellular acute myeloid leukemia is a rare entity and can affect young individuals. It can be considered as a rare cause of bone marrow necrosis.

## Introduction

The infrequent occurrence of hypocellularity at presentation of acute leukemia has been widely recognized. Hypocellular variants of acute leukemia almost always have a myeloid phenotype and usually develop secondary to radiation or chemotherapy [1,2]. They thus occur mainly in adults [2]. This paper describes two rare cases of hypocellular acute myeloid leukemia (AML) in young patients (a 32-year-old woman and a 13-year-old boy). Bone marrow necrosis (BMN) is a distinctive clinicopathologic entity characterized by necrosis of the medullary stroma and myeloid tissues [3]. Its etiology is diverse, and malignancy, especially hematopoietic in origin, is the most common underlying disease of BMN [4]. Hypocellular AML may be one of the important causes of BMN. Interestingly, in one of our patients we found grade 1 BMN.

## Case presentation

### Case 1

**Figure 1:**
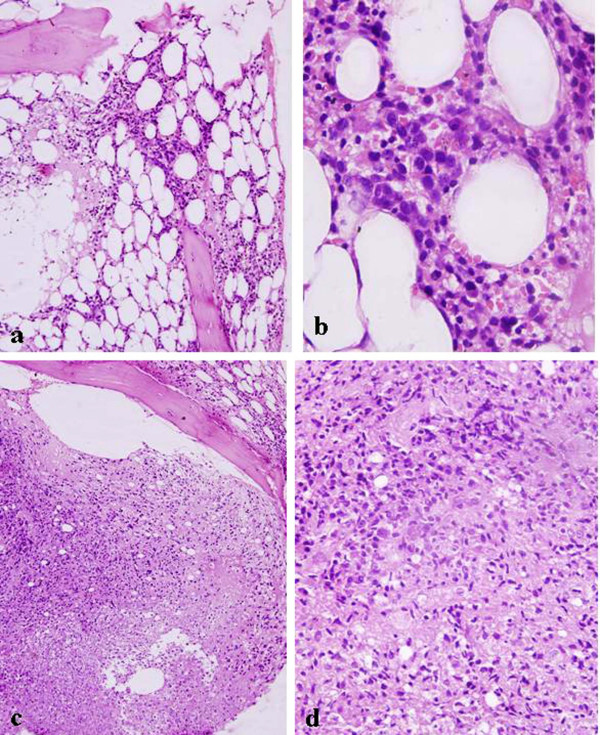
**Photomicrograph of bone marrow trephine biopsy (case 1) shows hypocellular marrow spaces with 70% of fat cells**. There are reduced numbers of hematopoietic cells with increased numbers of blasts (a, b). One of the focuses shows an area of bone marrow necrosis punctuated by hyperchromatic blasts and histiocytes (c, d). Hematoxylin and eosin stain, a ×40; b ×400; c ×40; d ×400.

A 32-year-old Indian woman presented with fever, generalized weakness, dyspnea on exertion and easy fatigability over a 3-month period. On examination, she had moderate pallor. There was no lymph node enlargement or organomegaly. Hematologic examination revealed hemoglobin of 5.3 g%, total leukocyte count of 9,100/mm^3^, and platelets of 15,000/mm^3^. No atypical cells were seen on peripheral blood smear examination. A clinical possibility of aplastic anemia was considered and bone marrow aspiration and biopsy were performed. Bone marrow aspirate smears were aparticulate and diluted with peripheral blood. However, there was an increase in the number of blasts. Correct blast enumeration was not possible due to dilution of aspirate by peripheral blood; however, blasts were the predominant cells. These blasts were large and had a scant to moderate amount of cytoplasm, opened-up chromatin and conspicuous nucleoli. Normal hematopoietic elements were markedly diminished. Cytochemically, these blasts were myeloperoxidase (MPO) positive. Based on all these features, a tentative diagnosis of acute myeloid leukemia French-American-British (FAB) subtype M1 (AML-M1) was made. Subsequently, trephine biopsy showed hypocellular marrow spaces with clusters of blasts in places. Erythroid series of cells were relatively preserved, however, myeloid and megakaryocytic lineages were markedly suppressed (Figure 1a, b). In addition, a focus of necrosis involving less than 20% of the biopsy area, along with scattered blasts was also seen at one edge of the biopsy (Figure 1c, d). Stains for acid fast bacilli and fungal organisms were negative, however, dispersed blasts were positive for anti-MPO stain. In view of the abovementioned findings, a diagnosis of hypocellular AML was established along with a focal area of necrosis. The patient was referred to a tertiary health care center for further management.

### Case 2

**Figure 2:**
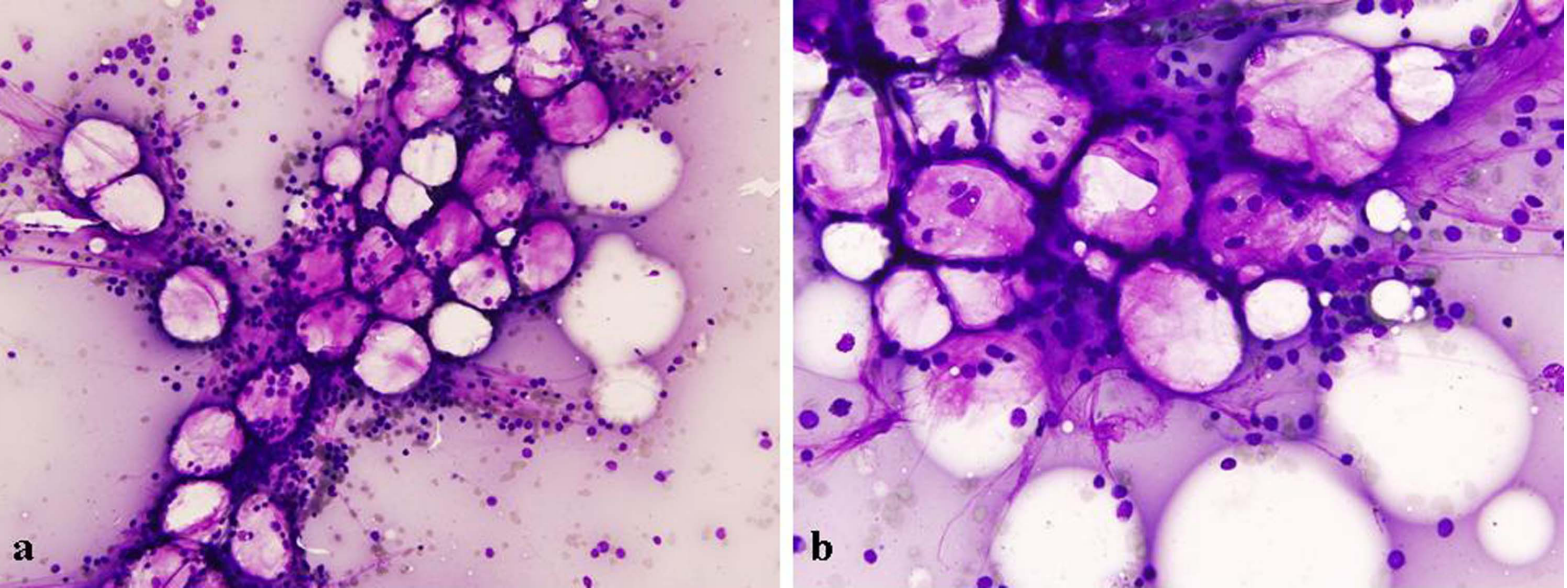
**Bone marrow aspirate smear (case 2) shows hypocellular marrow spaces with less than 50% cellularity and more than 30% blasts**. Wright Giemsa stain, a ×40; b ×600.

A 13-year-old Indian boy presented after a 10-day episode of right submandibular swelling. On examination, he had severe pallor and an enlarged hot and tender right submandibular gland. He had hepatomegaly. On ultrasonographic examination, enlarged mesenteric lymph nodes were identified. Hematologic examination revealed hemoglobin of 6.9 gm%, a total leukocyte count of 2,600/mm^3^, and platelet count of 35,000/mm^3^. Peripheral blood smear examination revealed 40% blasts with low platelets. These blasts were of intermediate size, had moderate cytoplasm, opened-up nuclear chromatin and 1 to 4 prominent nucleoli. Bone marrow aspirate and imprint smears were hypocellular for the age of the patient (less than 50% cellularity). There was an increased amount of fat, and marrow cells were replaced by blasts (88% of marrow cells) (Figure 2a, b). Normal hematopoietic elements were markedly diminished. Cytochemically, these blasts were myeloperoxidase positive. Trephine biopsy also displayed hypocellularity along with aggregates of blasts which were anti-MPO positive. In addition, foci of gelatinous marrow transformation were also identified. Finally, a diagnosis of hypocellular AML FAB subtype M1 was made. Chemotherapy was started, however, he succumbed 3 days after the diagnosis.

Unfortunately, clonal chromosomal abnormalities could not be evaluated in either patient due to unavailability of the facility in the department.

## Discussion

The occurrence of hypoplastic acute leukemia is widely recognized as an atypical leukemia, and is defined as hypocellular marrow with ≥20% blasts and none or few blasts in the circulating blood [5]. Clinically, it usually follows a less progressive course and has a high prevalence rate among the elderly. Although hypocellular acute lymphoblastic leukemias (ALL) almost always occur in children [2], to the best of our knowledge, hypocellular AML has not been reported in young and pediatric patients. There are many case series and individual case reports of hypoplastic acute leukemia available in the literature. Nagai *et al. *[1] proposed the following diagnostic criteria: pancytopenia with rare appearance of blasts in peripheral blood; less than 40% bone marrow hypocellularity; more than 30% blasts in bone marrow of all nucleated cells; and myeloid phenotypes of leukemic blasts by myeloperoxidase staining and/or immunophenotyping. Both of our patients fulfilled the abovementioned criteria except for the presence of 40% blasts in the peripheral smear in case 2. In both of our patients, almost all of the cells could be identified as blast cells, which made up more than 50% of nucleated marrow cells. FAB classification revealed a preponderance of the M1 category followed by M2 and M6 types [6]. We reported both of the cases as FAB M1 subtype, as there was less than 10% differentiation in the myeloid lineage and erythroid precursors were scarce. Moreover, there was no evidence of myelodysplasia. Beard *et al. *[7] and Needleman *et al. *[8] have reported their experience with hypoplastic acute leukemia and suggested that patients with hypocellular bone marrow experience a more indolent course, and can commonly achieve a good response to remission induction therapy. This disease has a proclivity for older patients, however, we found it in young patients. Although the majority of hypocellular acute leukemias are of myeloid type, rare case reports of hypocellular ALL are reported in the literature [9]. Hypocellular ALL usually presents in children but cases in elderly have been documented [9]. Recently, hypocellular acute promyelocytic leukemia with a tetraploid clone has also been reported [10]. The question of pathogenesis of the hypocellularity remains speculative. It is unclear whether the leukemia is secondary to the hypocellularity or if it is the primary event. It has been suggested that leukemia cell populations inhibit myelopoiesis through a humoral mechanism [11]. Alternatively, an increased susceptibility of myeloid precursors to the inhibitor in older patients might play a role in the genesis of hypoplasia [8]. However, the cause of hypoplasia in children needs to be elucidated. Although these patients appear to bear relatively low tumor cell burdens, the disease may pursue an aggressive course. In this report, case 2 died even after chemotherapy; unfortunately, we do not have follow-up of case 1. Recently, the beneficial effects of hematopoietic growth factors have been reported in the treatment of hypoplastic AML. It has been observed that chemotherapy may be necessary to maintain remission in hypoplastic AML after hematopoietic reconstitution by granulocyte colony stimulating factor (G-CSF) [12]. Although acute leukemia has been found to be the most common underlying cause of bone marrow necrosis [4], we did not find an association of BMN with hypoplastic AML in the literature. We have seen grade 1 BMN in case 1 along with scattered anti-MPO positive blasts. BMN was graded semiquantitatively according to the extent of necrosis in the bone marrow biopsy described by Maisel *et al. *[3].

## Conclusion

Hypoplastic acute myeloid leukemia is a distinct nosologic entity and can affect young individuals. It can be added to the growing body of literature as a rare cause of BMN.

## Abbreviations

ALL: acute lymphoblastic leukemia; AML: acute myeloid leukemia; BMN: bone marrow necrosis; G-CSF: granulocyte colony stimulating factor; MPO: myeloperoxidase

## Consent

Written informed consent was obtained from the patients for publication of this case report and any accompanying images. A copy of the written consent is available for review by the Editor-in-Chief of this journal.

## Competing interests

The authors declare that they have no competing interests.

## Authors' contributions

DJ drafted the manuscript and made the pathologic diagnoses while TS participated in the pathologic diagnoses. NK participated in the clinical evaluation of the patients.

## References

[B1] Nagai K Kohno T Chen YX Tsushima H Mori H Nakamura H Jinnai I Matsuo T Kuriyama K Tomonaga M Bennett JM Diagnostic criteria for hypocellular acute leukemia: a clinical entity distinct from overt acute leukemia and myelodysplastic syndrome Leuk Res 1996 20 563 574 10.1016/0145-2126(95)00136-0 8795690

[B2] Matloub YH Brunning RD Arthur DC Ramsay NK Severe aplastic anemia preceding acute lymphoblastic leukemia Cancer 1993 71 264 268 10.1002/1097-0142(19930101)71:1<264::AID-CNCR2820710140>3.0.CO;2-8 8416724

[B3] Maisel D Lim JY Pollock WJ Liu PI Bone marrow necrosis: an entity often overlooked Ann Clin Lab Sci 1998 18 109 115 3382156

[B4] Paydas S Ergin M Baslamisli F Yavuz S Zorludemir S Sahin B Bolat FA Bone marrow necrosis: clinicopathologic analysis of 20 cases and review of the literature Am J Hematol 2002 70 300 305 10.1002/ajh.10114 12210811

[B5] Vardiman JW Harris NL Brunning RD The World Health Organization (WHO) classification of the myeloid neoplasms Blood 2002 100 7 2292 2302 10.1182/blood-2002-04-1199 12239137

[B6] Tuzuner N Cox C Rowe JM Bennett JM Hypocellular acute myeloid leukemia: the Rochester (New York) experience Hematol Pathol 1995 9 195 203 8655464

[B7] Beard ME Bateman CJ Crowther DC Wrigley PF Whitehouse JM Fairley GH Scott RB Hypoplastic acute myelogenous leukaemia Br J Haematol 1975 31 167 176 10.1111/j.1365-2141.1975.tb00847.x 1059476

[B8] Needleman SW Burns CP Dick FR Armitage JO Hypoplastic acute leukemia Cancer 1981 48 1410 1414 10.1002/1097-0142(19810915)48:6<1410::AID-CNCR2820480624>3.0.CO;2-4 7272965

[B9] Kröber SM Horny HP Steinke B Kaiserling E Adult hypocellular acute leukaemia with lymphoid differentiation Leuk Lymphoma 2003 44 1797 1801 10.1080/1042819031000099661 14692536

[B10] Kojima K Imaoka M Noguchi T Narumi H Uchida N Sakai I Yasukawa M Fujita S Hypocellular acute promyelocytic leukemia with a tetraploid clone characterized by two t(15;17) Cancer Genet Cytogenet 2003 145 2 169 171 10.1016/S0165-4608(03)00097-9 12935930

[B11] Quesenberry PJ Rappeport JM Fountebouni A Sullivan R Zuckerman K Ryan M Inhibition of normal murine hematopoiesis by leukemic cells N Engl J Med 1978 299 2 71 75 351395 10.1056/NEJM197807132990204

[B12] Lee M Chubachi A Niitsu H Miura I Yanagisawa M Hirokawa M Miura AB Successful hematopoietic reconstitution with granulocyte colony-stimulating factor in a patient with hypoplastic acute myelogenous leukemia Intern Med 1995 34 692 694 10.2169/internalmedicine.34.692 7496088

